# Setting the research agenda for medically not yet explained symptoms (MNYES): a priority-setting partnership of patients, caregivers and clinicians

**DOI:** 10.1192/j.eurpsy.2023.452

**Published:** 2023-07-19

**Authors:** C. M. Van Der Feltz-Cornelis, J. Sweetman, A. Moriarty, P. Perros, A. Kaul, N. Gall

**Affiliations:** ^1^Health Sciences, University of York, York; ^2^Institute of Human Genetics, University of Newcastle upon Tyne, Newcastle upon Tyne; ^3^ St. George’s University Hospitals NHS Foundation Trust; ^4^King’s College Hospital, King’s College, London, United Kingdom

## Abstract

**Introduction:**

This study establishes research priorities for Medically Not Yet Explained Symptoms (MNYES). A significant number of patients suffer from these symptoms, also known as MUS, that are likely to cause work disability and impact on quality of life. Research into MNYES in general has been poorly funded over the years, has been primarily researcher-led, and was sometimes controversial.

**Objectives:**

To identify research priorities from the perspective of patients, caregivers and clinicians, following the James Lind Alliance (JLA) priority setting partnership (PSP) method.

**Methods:**

The PSP Steering Group termed these symptoms Medically Not Yet Explained Symptoms (MNYES). This was an operational definition not intended to add to or replace other definitions already in use, that was constructed to embrace the views of all stakeholders. The nomenclature MNYES was chosen to indicate our incomplete understanding of these conditions. This could pertain to biological, psychological and social factors, as well as factors involving the trajectory of patients through various healthcare settings.

The study involved five key stages: defining the appropriate term for the conditions under study by the PSP Steering Group; gathering questions on MNYES from patients, caregivers and clinicians in a publicly accessible survey; checking these research questions against existing evidence; interim prioritisation in a second survey; and a final multi-stakeholder consensus meeting to determine the top 10 unanswered research questions using the modified nominal group methodology.

**Results:**

Over 700 responses from UK patients, caregivers and clinicians were identified in two surveys from a broad range of medical specialities and primary care. Patients prioritised research questions regarding diagnosis and aetiology; clinicians and caregivers prioritised outcomes and treatment, relatively.

The top 10 unanswered research questions cover the domains of: treatment; the role of clinicians; symptoms and outcomes; and recovery.

**Conclusions:**

This JLA PSP may well be the first attempt at capturing the thoughts of a wide group of medical professionals, patients and caregivers in one place with the aim eventually of standardising care and reducing unhelpful variability in the management of MNYES. Following the JLA approach is a strength of the study. The choice of the term MNYES conveys a message of hope, which responds to a need identified by patients, carers and clinicians alike for vigorous research in this domain. The research priorities are expected to generate much-needed, relevant and impactful research into MNYES. Better funding possibilities for MNYES are urgently needed.

**Disclosure of Interest:**

None Declared

